# Plasmidome in *mcr-1* harboring carbapenem-resistant enterobacterales isolates from human in Thailand

**DOI:** 10.1038/s41598-022-21836-7

**Published:** 2022-11-09

**Authors:** Parichart Boueroy, Thidathip Wongsurawat, Piroon Jenjaroenpun, Peechanika Chopjitt, Rujirat Hatrongjit, Sathaporn Jittapalapong, Anusak Kerdsin

**Affiliations:** 1 Faculty of Public Health, Kasetsart University Chalermphrakiat Sakon Nakhon Province Campus, Sakon Nakhon, 47000 Thailand; 2 Division of Bioinformatics and Data Management for Research, Department of Research and Development, Faculty of Medicine Siriraj Hospital, Mahidol University, Bangkok, Thailand; 3 Department of General Sciences, Faculty of Science and Engineering, Kasetsart University Chalermphrakiat Sakon Nakhon Province Campus, Sakon Nakhon, 47000 Thailand; 4 Department of Parasitology, Faculty of Veterinary Medicine, Kasetsart University, Chatuchak, Bangkok, 10900 Thailand

**Keywords:** Microbiology, Molecular biology

## Abstract

The emergence of the mobile colistin-resistance genes *mcr-1* has attracted significant attention worldwide. This study aimed to investigate the genetic features of *mcr-1*-carrying plasmid among carbapenem-resistant Enterobacterales (CRE) isolates and the potential genetic basis governing transmission. Seventeen *mcr*-harboring isolates were analyzed based on whole genome sequencing using short-read and long-read platforms. All the *mcr-1*-carrying isolates could be conjugatively transferred into a recipient *Escherichia coli* UB1637. Among these 17 isolates, *mcr-1* was located on diverse plasmid Inc types, consisting of IncX4 (11/17; 64.7%), IncI2 (4/17; 23.53%), and IncHI/IncN (2/17; 11.76%). Each of these exhibited remarkable similarity in the backbone set that is responsible for plasmid replication, maintenance, and transfer, with differences being in the upstream and downstream regions containing *mcr-1*. The IncHI/IncN type also carried other resistance genes (*bla*_TEM-1B_ or *bla*_TEM-135_). The *mcr-1*-harboring IncX4 plasmids were carried in *E. coli* ST410 (7/11; 63.6%) and ST10 (1/11; 9.1%) and *Klebsiella pneumoniae* ST15 (1/11; 9.1%), ST336 (1/11; 9.1%), and ST340 (1/11; 9.1%). The IncI2-type plasmid was harbored in *E. coli* ST3052 (1/4; 25%) and ST1287 (1/4; 25%) and in *K. pneumoniae* ST336 (2/4; 50%), whereas IncHI/IncN were carried in *E. coli* ST6721 (1/2; 50%) and new ST (1/2; 50%). The diverse promiscuous plasmids may facilitate the spread of *mcr-1* among commensal *E. coli* or *K. pneumoniae* strains in patients. These results can provide information for a surveillance system and infection control for dynamic tracing.

## Introduction

The global spread of carbapenem-resistant Enterobacterales (CRE) has become a leading public health concern due to the rapidly increasing prevalence of carbapenemase gene carriage by Enterobacterales, with most carbapenem resistance conferred by carbapenem-degrading enzymes (carbapenemase) such as *K. pneumoniae* carbapenemase (*bla*_KPC_), New Delhi metallo-β-lactamase (*bla*_NDM_), and OXA-48-like carbapenemase^1,2^.

The lack of accessible treatment has resulted in the use of colistin, an outmoded antibiotic, as a last-resort therapeutic drug for human infections by Gram-negative bacteria. The widespread use of colistin in humans and animals has led to the emergence of colistin resistance in Gram-negative bacteria, with rates of resistance continuously increasing^3,4^. A classic mechanism of colistin resistance is thought to be associated with chromosomal mediation^5^. The discovery of plasmid-mediated colistin resistance encoded by *mcr* genes revealed high prevalence in human and animal isolates harboring these genes and the transmission of *mcr* is of global concern^6^. Up to the present, 10 variants of *mcr* (*mcr-1* through *mcr-10*) have been reported^7,8^. Of particular concern is the spread of *mcr* genes into CRE, which would create strains that are potentially pan-drug resistant. The coexistence of *mcr* and carbapenemase genes, such as *bla*_NDM_, *bla*_OXA-48-like_, and *bla*_IMP_, in CRE isolates has been described worldwide^9–12^.

The global prevalence of *mcr* genes revealed that *mcr-1* (4917/5191; 94.7%) is a common gene and has a wider distribution than *mcr*-*2* through to *mcr-8*^4^. Human infections with CRE isolates carrying *mcr-1* have been reported^10–14^ and the prevalence of *mcr-1* has been increasing in Thailand^13^. The *mcr*-carrying plasmids identified consist of IncX4, IncI2, IncHI2, IncF, IncP, IncY, and ColE10-like ones, most of which are conjugative plasmid^15^.

Collectively, information regarding the genetic context of *mcr-1* plasmid and its organization in the genome is still limited in Thailand. One study revealed the general characteristics of *mcr-1* harboring CRE isolated from patients in Thailand^13^. However, genomic analysis has not yet determined insights to plasmidome in the CRE harboring *mcr-1*. Thus, this study aimed to determine the complete genomic sequences to provide insight into plasmidome and to compare plasmid harboring *mcr-1*- among CRE isolates from human patients in Thailand.

## Materials and methods

### Bacterial isolates

This study used 17 CRE carrying *mcr-1* isolates, consisting of 12 isolates (*E. coli* = 8; *K. pneumoniae* = 4)) in a previous study^13^ and 5 isolates (*E. coli* = 4; *K. pneumoniae* = 1) sent by hospitals in Thailand for further confirmation by the Public Health Microbiological Laboratory of the Faculty of Public Health, Kasetsart University Chalermphrakiat Sakon Nakhon Province Campus under the Emerging Antimicrobial Resistant Bacterial Surveillance Program (EARB) during 2016–2019 (Table 1). The presence of *mcr-1*–*mcr-9*^16^ and carbapenemase genes (*bla*_IMP_, *bla*_KPC_, *bla*_NDM_, and *bla*_OXA‐48‐like_)^17^ was confirmed in these CRE isolates using Multiplex polymerase chain reaction (PCR), as previously described.

**Table 1:**
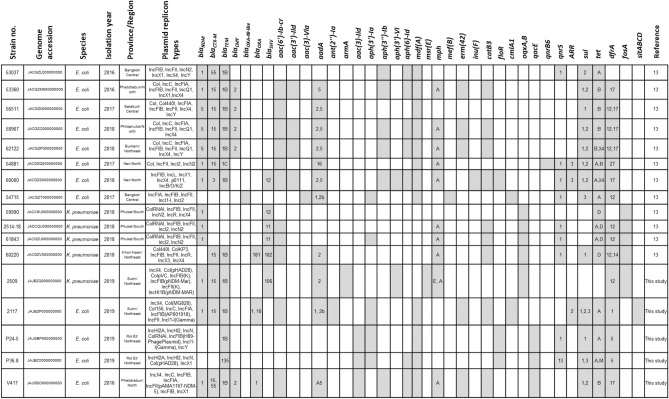
Distribution of sequence types and antimicrobial-resistant genes in *mcr-1*-carrying *E. coli* and *K. pneumoniae* strains.

### Ethical approval

Ethical review and approval were not required because no human specimens or data were used in the current study.

### Antimicrobial susceptibility testing

The minimal inhibitory concentration (MIC) of colistin was determined in 5 CRE carrying *mcr-1* isolates using the broth microdilution method according to 2021 Clinical and Laboratory Standards Institute guidelines^18^. The broth microdilution method was conducted using cation-adjusted Mueller–Hinton broth (Becton, Dickinson and Company, Sparks, MD, USA). MIC values ≤ 2 µg/ml were interpreted as intermediate susceptibility, whereas an MIC of ≥ 4 µg/ml was considered resistant. Antimicrobial susceptibility to ampicillin, gentamicin, amikacin, amoxicillin/clavulanic acid, amoxicillin/sulbactam, pipercillin-tazobactam, trimethoprime/sulfamethoxazole, cefepime, cefotaxime, ciprofloxacin, levofloxacin, ertapenem, imipenem, meropenem, doripenem, ceftazidime, ceftriaxone, cefoxitin, and netilmicin was performed with a Vitek® 2 automated system (Clinical Microbiology Laboratory, Sakon Nakhon Hospital).

### Conjugation assay

Conjugation assay was performed in all 17 *mcr-1*-carrying CRE isolates consisting of *E. coli* (n = 12) and *K. pneumoniae* (n = 5) isolates, as previously described^19,20^. The *mcr-*harboring CRE strains (donor) and streptomycin-resistant *E. coli* UB1637 (recipient) were mixed in a ratio of 1:25. The mixtures were collected and then plated on MacConkey agar containing streptomycin (3200 μg/ml) and colistin (4 μg/ml). The transconjugants harboring *mcr* genes were confirmed using PCR^16^.

### Complete genome sequencing

Bacterial genomic DNA samples were extracted using ZymoBIOMICS DNA Kits (Zymo Research, CA, USA) according to manufacturer’s instructions. Only 12 isolates from the previous study were sequenced by Oxford Nanopore Technologies (ONT)^13^, while 5 isolates were sequenced using the ONT and Illumina platforms. Library preparation for ONT sequencing followed the rapid barcoding DNA sequencing protocol with the SQK-RBK004 kit without DNA size selection (to preserve the plasmid DNA) and the libraries were sequenced using a single R9.4.1/FLO-MIN106 flow cell on a MinION Mk1B sequencer. We base-called and demultiplexed the raw data using Guppy v3.4.5 (ONT), specifying the high-accuracy model (-c dna_r9.4.1_450bps_hac.cfg). The ONT adapters were trimmed using Porechop v0.2.4 (https://github.com/rrwick/Porechop). Quality control of ONT reads was undertaken using Nanoplot v1.28.1 (https://github.com/wdecoster/NanoPlot).

For the Illumina platform, the sequencing library was generated using a NEBNext Ultra II DNA Library Prep Kit for Illumina (New England Biolabs, UK), following the manufacturer’s recommendations. The genomic DNA was randomly fragmented to a size of 350 bp and the fragments were A-tailed and ligated with the adapter. Libraries were sequenced using the Illumina HiSeq platform with the 150 paired-end sequencing strategy. We applied Fastp v0.19.5^21^ with default parameters for the quality filtering of Illumina reads. Adapters were trimmed using Skewer v0.2.2^22^. The quality checking of Illumina reads was performed using FastQC v0.11.8 (https://www.bioinformatics.babraham.ac.uk/ projects/fastqc/). Hybrid assemblies with the ONT and Illumina data were performed using Unicycler v0.4.8^23^ and the genome sequences of all 17 isolates were checked for quality using QUAST v5.0.2^24^. Genome sequences were submitted to the NCBI Prokaryotic Genome Annotation Pipeline (PGAP v4.12) for annotation. The default parameters were used for all software unless otherwise specified.

### Bioinformatics analysis

Identification of antimicrobial resistance genes was analyzed using ResFinder 4.1^25^ and the Comprehensive Antibiotic Resistance Database (CARD)^26^. Determination of the *mcr-1*-carrying plasmid was carried out using PlasmidFinder^27^. Phylogrouping for *E. coli* and the KL type of *K. pneumoniae* were performed using ClermonTyping^28^ and Kaptive^29^, respectively. Multilocus sequence typing (MLST) analysis of *mcr-1*-carrying *E. coli* and *K. pneumoniae* was determined using MLST 2.0^30^.

To search for the genetically closest relatives to the *mcr*-carrying isolates, a modular single genome analysis was conducted following the core genome multilocus sequence typing approach by BacWGSTdb 2.0^31^. The genetically closest relatives were chosen for 5–10 strains based on small numbers of allelic differences with selection thresholds of 100–500, depending on the strains under current study. The phylogenetics of the *mcr*-carrying CRE isolates and the closest relatives selected from BacWGSTdb were conducted using a reference genome-based, single-nucleotide polymorphism (SNP) strategy with REALPHY^32^. The phylogenetic tree was visualized using the iTOL V4 software^33^. *E. coli* K12 substrain MG1655 (accession no. U00096) and *K. pneumoniae* WCHKP9G2 (accession no. NBYD01000091) were used as the reference sequences for SNP analysis. In addition, phylogenetic analysis of plasmid-harboring *mcr-1* was conducted using the Mashtree program, following the program’s instructions^34^.

### Accession number

The assembled genomic sequences were deposited under the BioProject accession number PRJNA525849. The accession numbers for each *mcr-1*-harboring isolate are provided in Table 1.

## Results

### Antimicrobial susceptibility of* mcr-1*-harboring CRE isolates

In a previous study, 4516 (64.5%) CRE were identified from 6996 multidrug-resistant isolates. Of these, 4235 (93.7%) isolates were classified as carbapenemase-producing Enterobacterales (CPE) and carried carbapenemase genes including *bla*_NDM_, *bla*_OXA-48-like_, *bla*_IMP_, or coexisting carbapenemase genes according to the modified carbapenem inactivation method (mCIM) and PCR results^13^. Of all the CPE isolates*,* 13 (0.3%) carried *mcr* genes^13^. That study did not detect *mcr-1* in other species of Enterobacterales except *E. coli* and *K. pneumoniae*^13^. In the current study, five additional Enterobacterales isolates from the EARB program were included. Unfortunately, one isolate of the previous study was unrecovered. Therefore, a total of 17 isolates were conducted to their completed genome and further analysis.

**Figure 1:**
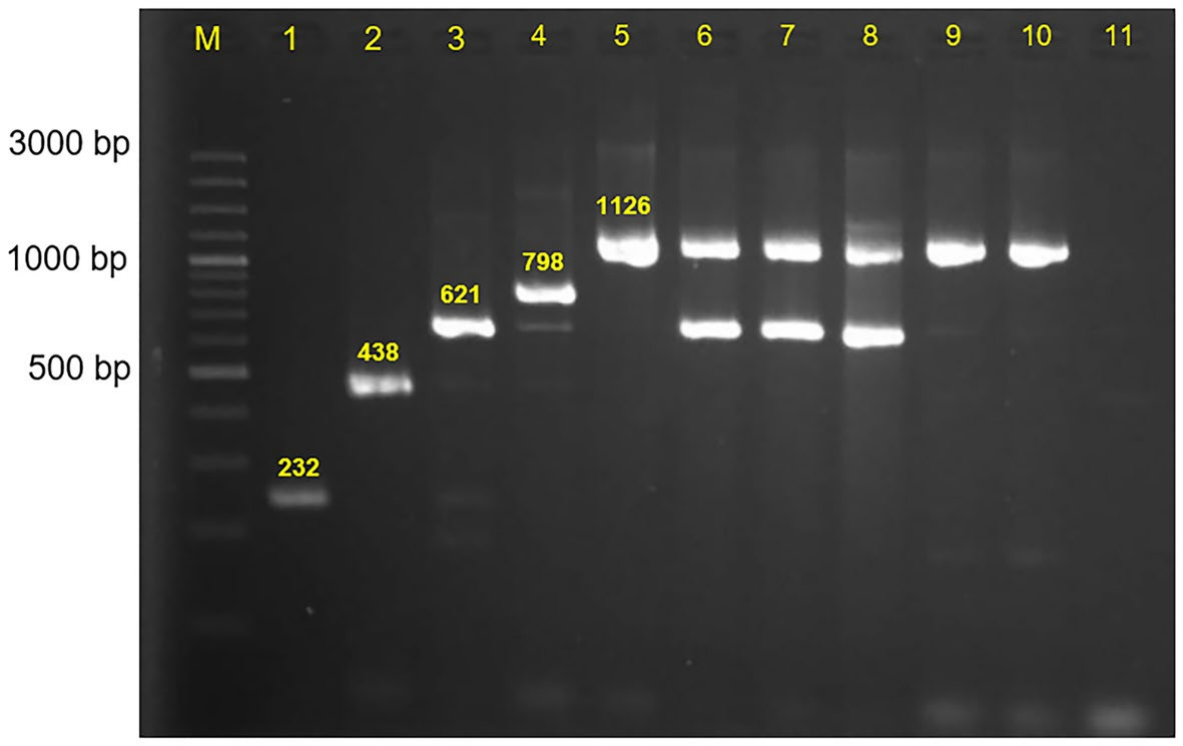
Agarose gel electrophoresis of PCR-amplified products of carbapenemase and *mcr-1* genes from five *mcr-1*-carrying *E. coli* and *K. pneumoniae* isolates. Positive control of *bla*_IMP (lane 1),_ *bla*_OXA-48-like (lane 2),_ *bla*_NDM (lane 3),_ *bla*_KPC (lane 4),_ *mcr-1* (lane 5), *K. pneumoniae* strain no. 2509 (lane 6), *E. coli* strain no. 2117 (lane 7), V417 (lane 8), P24-5 (lane 9), and P36.8 (lane 10), negative control (lane 11). A 100-bp DNA ladder is shown in lane M.

PCR confirmed the presence of *mcr-1* in all 17 isolates, with one isolate (no. 54715) coexisting with *mcr-3*. Five additional *mcr-1*-carrying isolates in the current study presented carbapenemase genes including *bla*_NDM-1_ in three isolates (strain nos. 2509, 2117, and V417), whereas in two isolates (strain nos. P24-5 and P36.8) the carbapenemase genes were not detected (Fig. 1). Only five isolates carrying *mcr-1* isolates included in this study were determined for antimicrobial susceptibility because the other 12 CRE isolates had already been described elsewhere^13^. All 17 *mcr-1*-harboring *E. coli* (n = 12) and *K. pneumoniae* (n = 5) isolates were resistant to colistin (MIC values 4–16 µg/ml), ampicillin and ampicillin/sulbactam (Table 2). Fifteen isolates (88.24%) of the *mcr-1*-harboring strains were resistant to carbapenems. Among the five isolates carrying *mcr-1* isolates included in this study, three were resistant to ampicillin, ampicillin/sulbactam, Piperacillin/tazobactam, cefepime, cefotaxime, cefoxitin, imipenem, meropenem, ertapenem, ciprofloxacin, and levofloxacin. Two *mcr-1-*carrying *E. coli* isolates (P24-5 and P36.8) were resistant only to ampicillin, ampicillin/sulbactam, gentamicin, levofloxacin, and ciprofloxacin whereas they were susceptible to the carbapenems (Table 2).

**Table 2:** Antimicrobial susceptibility of *mcr-1*-harboring carbapenem-resistant *E. coli* and *K. pneumoniae* strains.

Isolate No		53360	54881	56511	58967	62122	60000	54715	53037	59990	60220	61843	2414–18	2509	2117	P24.5	P36.8	V417
Oganism		E. *coli*	*E. coli*	*E. coli*	*E. coli*	*E. coli*	*E. coli*	*E. coli*	*E. coli*	*K. pneumoniae*	K. *pneumoniae*	*K. pneumoniae*	*K. pneumoniae*	*E. coli*	*K. pneumoniae*	*E. coli*	*E. coli*	*E. coli*
Reference		^13^	^13^	^13^	^13^	^13^	^13^	^13^	^13^	^13^	^13^	^13^	^13^	This study	This study	This study	This study	This study
Penicillin	Ampicillin	> 256 R	> 256 R	> 256 R	> 256 R	> 256 R	> 256 R	> 256 R	> 256 R	> 256 R	> 256 R	> 256 R	> 256 R	> 32 R	> 16 R	> 16 R	> 16 R	> 16 R
β-lactam	Amoxicillin/Clavulanic Acid	> 256 R	> 256 R	> 256 R	> 256 R	> 256 R	> 256 R	> 256 R	> 256 R	> 256 R	> 256 R	> 256 R	> 256 R	> 16 R	> 16 R	16 I	16 I	> 16 R
Combination	Ampicillin/Sulbactam	> 256 R	> 256 R	> 256 R	> 256 R	> 256 R	> 256 R	> 256 R	> 256 R	> 256 R	> 256 R	> 256 R	> 256 R	> 16 R	> 16 R	> 16 R	> 16 R	> 16 R
	Piperracillin/Tazobactam	> 256 R	> 256 R	> 256 R	> 256 R	> 256 R	> 256 R	64 I	> 256 R	> 256 R	> 256 R	> 256 R	> 256 R	> 64 R	> 64 R	32 I	16 S	> 64 R
3rd generation Cephalosporin	Cefepime	> 256 R	> 256 R	> 256 R	> 256 R	> 256 R	> 256 R	0.125 S	> 256 R	> 256 R	> 256 R	96 R	96 R	> 32 R	> 32 R	< = 1 S	< = 1 S	> 32 R
	Cefotaxime	> 32 R	> 32 R	> 32 R	> 32 R	> 32 R	> 32 R	0.25 S	> 32 R	> 32 R	> 32 R	> 32 R	> 32 R	> 32 R	> 32 R	< = 1 S	< = 1 S	> 32 R
	Cefoxitin	> 256 R	> 256 R	> 256 R	> 256 R	> 256 R	> 256 R	8 S	> 256 R	> 256 R	> 256 R	> 256 R	> 256 R	> 16 R	> 16 R	> 16 R	16 I	> 16 R
Ca rbapenems	Imipenem	> 32 R	> 32 R	1.5 I	8 R	8 R	> 32 R	0.25 S	4 R	32 R	> 8 R	> 32 R	> 32 R	> 16 R	16 R	< = 0.5 S	< = 0.5 S	> 16 R
	Meropenem	> 32 R	> 32 R	12 R	12 R	8 R	> 32 R	0.5 S	8 R	> 32 R	> 8 R	> 32 R	> 32 R	> 16 R	> 16 R	< = 0.5 S	< = 0.5 S	> 16 R
	Ertapenem	> 32 R	> 32 R	> 32 R	> 32 R	> 32 R	> 32 R	0.75 I	> 32 R	> 32 R	> 4 R	> 32 R	> 32 R	> 4 R	> 4 R	< = 0.5 S	< = 0.5 S	> 4 R
Aminoglycoside	Amikacin	32 I	4 S	8 S	6 S	3 S	3 S	2 S	3 S	2 S	32 I	4 S	6 S	> 32 R	< = 8 S	< = 8 S	< = 8 S	< = 8 S
	Gentamicin	64 R	32 R	0.75 S	32 R	16 R	> 256 R	24 R	0.75 S	0.5 S	0.75 S	0.75 S	0.75 S	> 8 R	8 I	> 8 R	> 8 R	> 8 R
Quinolone	Levofloxacin	> 32 R	> 32 R	> 32 R	> 32 R	> 32 R	> 32 R	> 32 R	> 32 R	0.125 S	> 32 R	0.5 S	> 32 R	4 R	> 8 R	4 R	2 R	> 8 R
	Ciprofloxacin	> 32 R	> 32 R	> 32 R	> 32 R	> 32 R	> 32 R	> 32 R	> 32 R	0.094 S	> 32 R	1.5 R	1 R	> 2 R	> 2 R	2 R	1 R	> 2 R
Colistin	Colistin	4 R	4 R	4 R	4 R	4 R	8 R	4 R	8 R	8 R	8 R	16 R	16 R	> 8 R	8 R	> 8 R	> 8 R	8 R

### Conjugative transfer of the *mcr-1* gene

**Figure 2:**
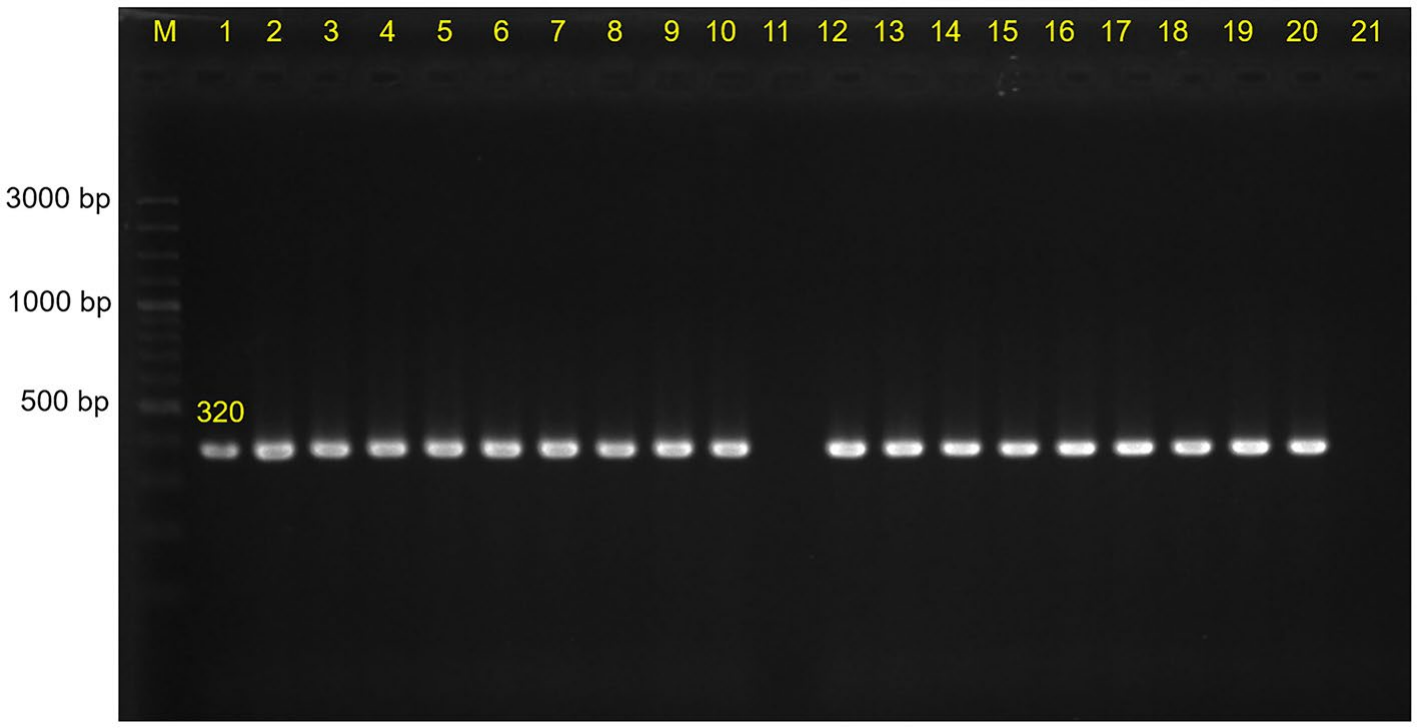
Agarose gel electrophoresis of PCR-amplified products from the transconjugants *E. coli* UB1637**.** Conjugation assay was performed in *mcr-1*-carrying CRE isolates. The transconjugants were collected nine colonies in each sample and confirmed the *mcr-1* gene using PCR. Positive control (lane 1), *E. coli* strain no. 58967 (lane 2–10), negative control (lane 11), *E. coli* strain no. 56511 (lane 12–20), negative control (lane 22). A 100-bp DNA ladder is shown in lane M.

As shown in Fig. 2 and Table 3, all 17 *mcr-1*-carrying CRE isolates could be conjugatively transferred into *E. coli* UB1637. One coexisted of *mcr-1* and *mcr-3 E. coli* (isolate no. 54715), transferring both. Colistin resistance was also detected in 14 *E. coli* recipient (82.35%) with MIC values in the range 4–16 µg/ml, whereas the rest were susceptible (Table 3). This suggested that *mcr-1* in all 17 CRE isolates carried on the conjugative plasmids.

**Table 3:** Profiles of antimicrobial-resistance genes of donors *E. coli*, *K. pneumoniae* and tranconjugants.

Code	Strain	*mcr*	MIC	Donor	Tranconjugant
		Donor	Tranconjugant		
53037	*E. coli*	*mcr-1*	*mcr-1*	8	8
53360	*E. coli*	*mcr-1*	*mcr-1*	8	8
54715	*E. coli*	*mcr-1, mcr-3*	*mcr-1, mcr-3*	8	8
54881	*E. coli*	*mcr-1*	*mcr-1*	8	8
56511	*E. coli*	*mcr-1*	*mcr-1*	8	8
58967	*E. coli*	*mcr-1*	*mcr-1*	8	8
59990	*K. pneumoniae*	*mcr-1*	*mcr-1*	8	8
60000	*E. coli*	*mcr-1*	*mcr-1*	4	16
60220	*K. pneumoniae*	*mcr-1*	*mcr-1*	16	16
61843	*K. pneumoniae*	*mcr-1*	*mcr-1*	16	8
62122	*E. coli*	*mcr-1*	*mcr-1*	8	16
2514-18	*K. pneumoniae*	*mcr-1*	*mcr-1*	16	8
2117	*E. coli*	*mcr-1*	*mcr-1*	8	2
2509	*K. pneumoniae*	*mcr-1*	*mcr-1*	> 8	4
P24.5	*E. coli*	*mcr-1*	*mcr-1*	> 8	1
P36.8	*E. coli*	*mcr-1*	*mcr-1*	> 8	1
V417	*E. coli*	*mcr-1*	*mcr-1*	8	16

### Genomic characterization of *mcr*-harboring CRE isolates

Table 1 summarizes the antimicrobial-resistant genes in all 17 *mcr-1* carrying isolates. Five additional *mcr-1*-carrying isolates in the current study presented an associated β-lactamase gene: *bla*_CTX-M-15_, *bla*_CTX-M-55_, *bla*_SHV-106_, *bla*_CMY-2_, *bla*_TEM-1B,_ and *bla*_TEM-135_. Of these, three isolates were coexisting carbapenemase genes including *bla*_NDM-1_ (Table 1 and Fig. 1). Other antimicrobial-resistance genes in the five isolates are shown in Table 1. Finally, β-lactamase-encoding genes in *mcr*-carrying isolates were located on different plasmid replicon types: IncFIA, IncFIB, IncFII, IncC, or IncI1-I (Table 1).

Based on MLST analysis, we detected 4 different STs in five additional *mcr-1* carrying isolates; ST410, ST15, ST6726, and new ST (Table 1). Clermont phylotyping of four *mcr*-harboring *E. coli* isolates showed phylogroups C (2/4; 50%), A (1/4; 25%), and Clade III (1/4; 25%) while eight *E. coli* isolates in the previous study revealed 5 phylogroup C and 2 and 1 for phylogroups A and D, respectively^13^. We concluded that the predominant phylogroup in all 17 isolates was C, accounting for 41.18% (7/17).

**Figure 3:**
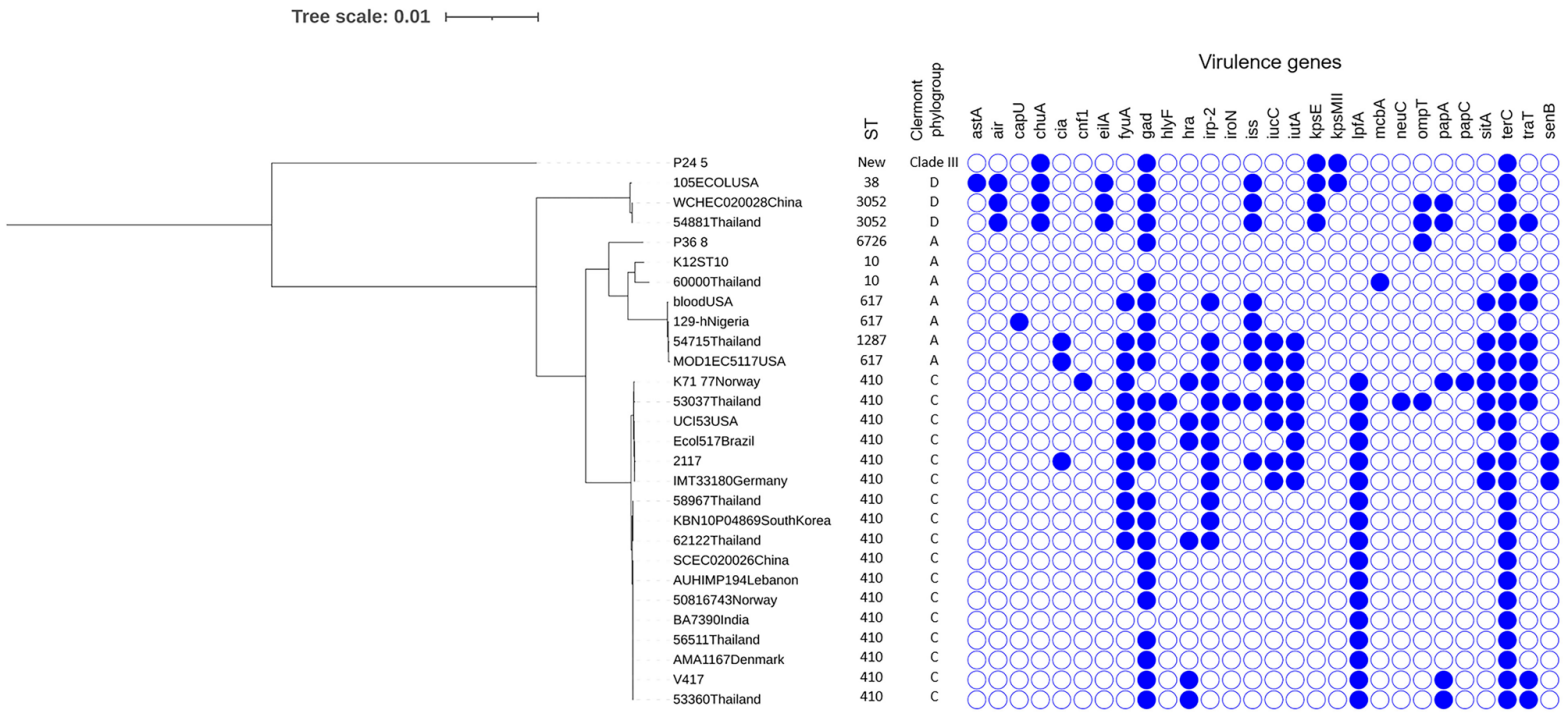
Phylogenetic tree based on single nucleotide polymorphisms (SNP) using the neighbor-joining method, sequence types (STs) and virulence gene patterns in *E. coli*. Virulence genes are represented by respective blue-colored shapes. The tree was visualized and annotated using Interactive Tree of Life (iToL).

All 12 *mcr-1*-harboring *E. coli* isolates carried the virulence genes *gad* (glutamate decarboxylase) and *terC* (tellurium ion resistance protein). Five *mcr-1*-harboring *K. pneumoniae* isolates carried *fyuA* (siderophore receptor), *iutA* (ferric aerobactin receptor), and *irp2* (iron regulatory protein), as shown in Fig. 3. An additional single *K. pneumoniae* isolate included in the current study was KL type 28, whereas 3 KL25 and 1 KL15 were detected in *K. pneumoniae* in the previous study^13^.

**Figure 4:**
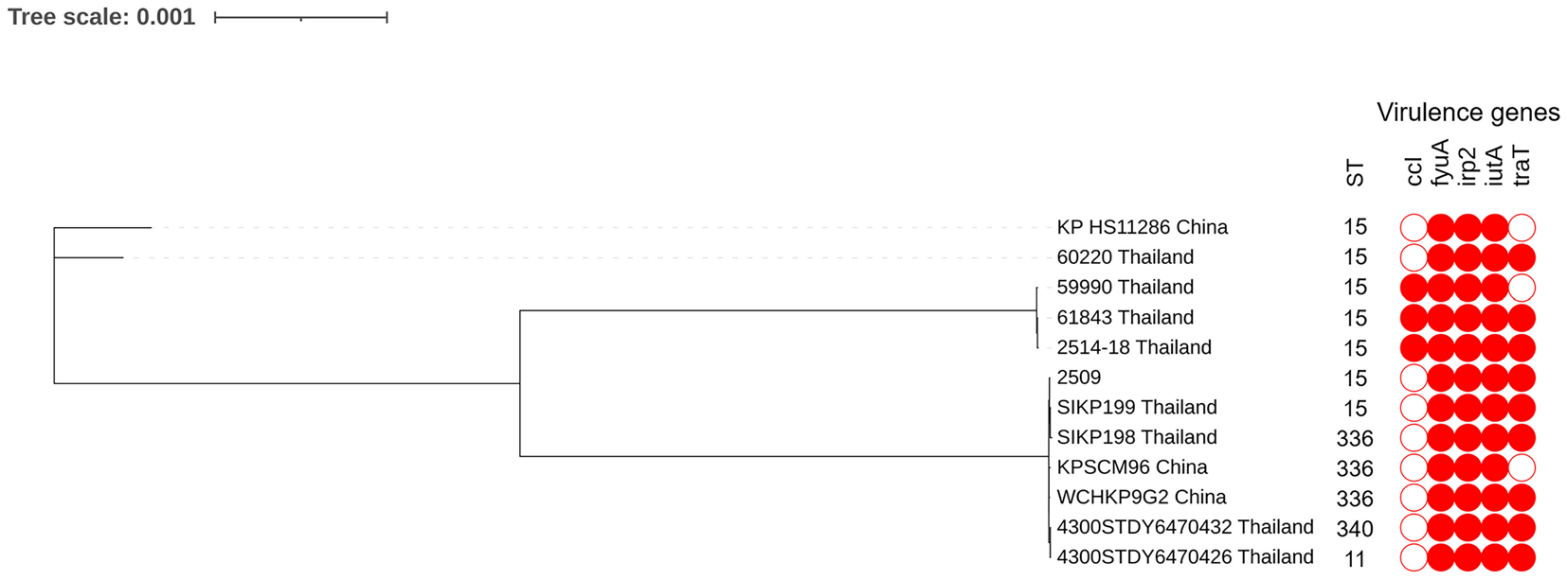
Phylogenetic tree based on SNP, STs, and virulence gene patterns in *K. pneumoniae*. Virulence genes are represented by respective red-colored shapes. The tree was visualized and annotated using iToL.

The genetic relationships based on the SNPs phylogeny of these *mcr-1*-harboring isolates are shown in Fig. 3 and Fig. 4. *E. coli* strain no. 2117 was closely related with strains from China (accession no. CP035123.1). Isolate no. P36.8 was closely related with the reference strain K12 and clustered with P24-5 (new ST), as shown in Fig. 3. *K. pneumoniae* strain no. 2509 was closely related with the *K. pneumoniae* SIKP199 strain from Thailand (accession no. GCA_004833525.1) (Fig. 4).

### Analysis of *mcr-1*-bearing plasmids

**Figure 5:**
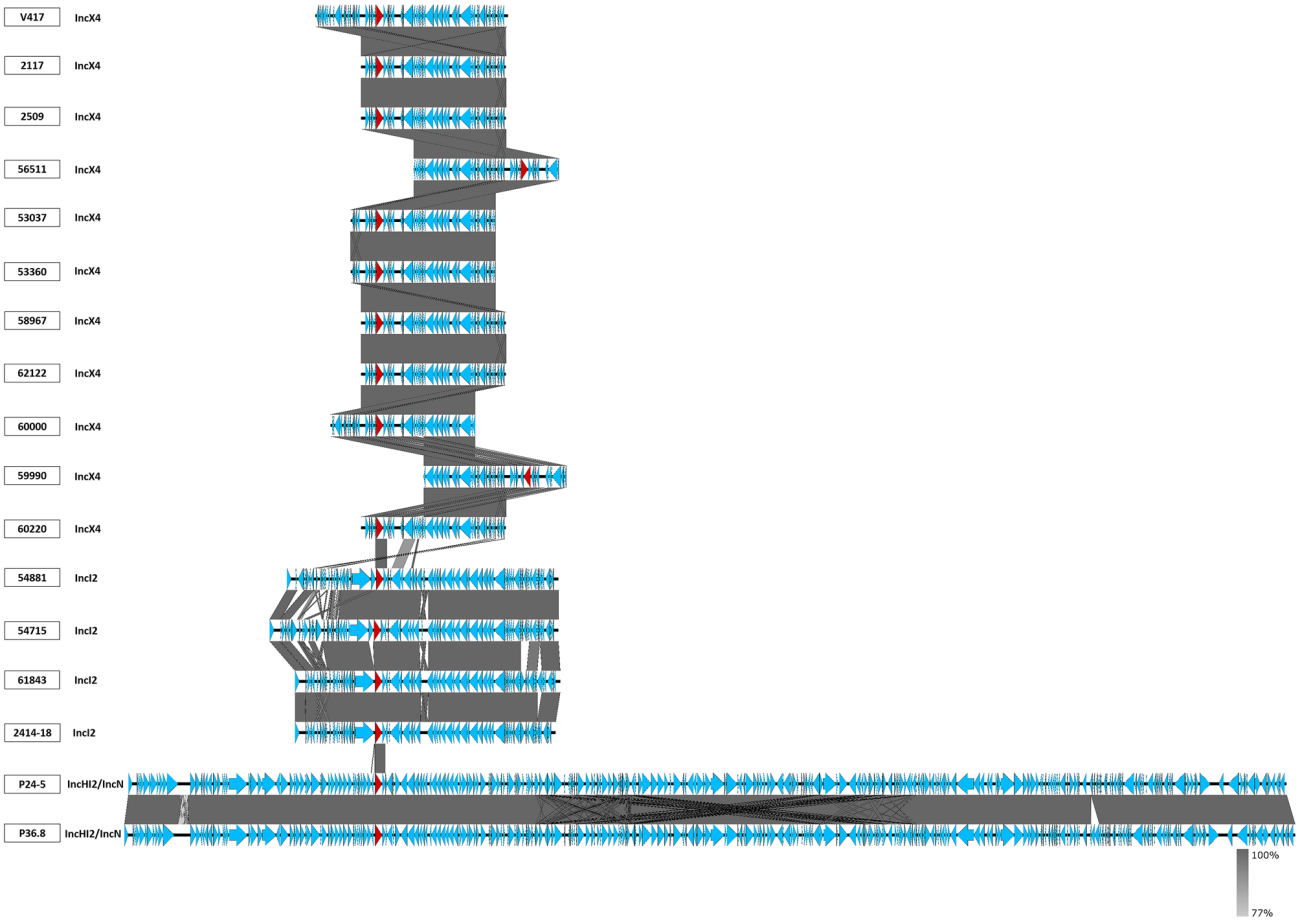
Alignment of plasmids carrying *mcr-1* genes. Horizontal red arrows indicate location, size, direction of transcription, and orientation of open reading frames. The red color code indicates *mcr-1*. Homologous segments generated by a BLASTn comparison are shown as gray blocks that are connected across plasmids.

**Figure 6:**
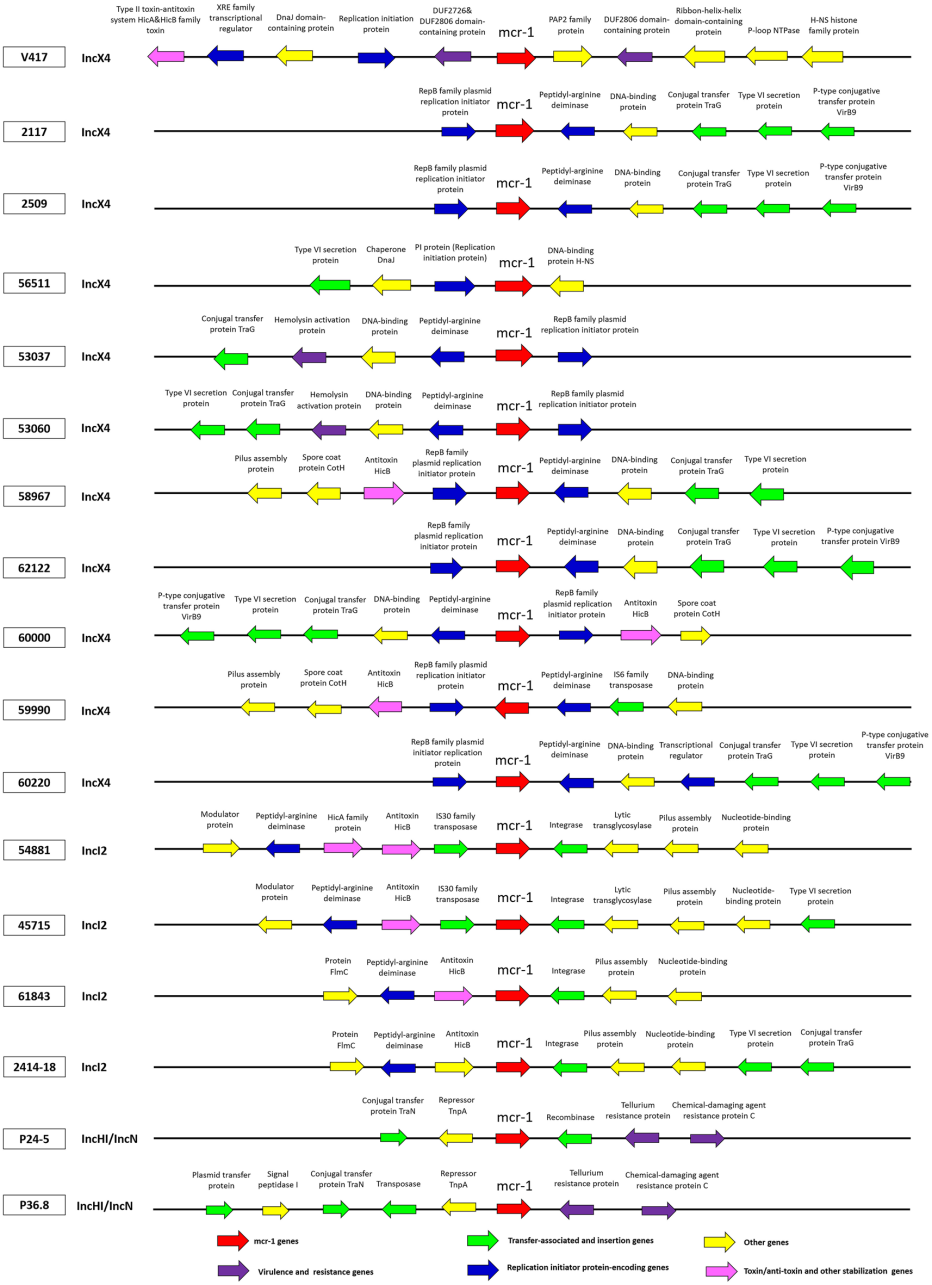
Schematic partial representation of coding sequences or genes surrounding *mcr-1* among 17 *mcr*-carrying plasmids in *E. coli* and *K. pneumoniae*. The coding sequences are represented by arrows pointing toward their respective orientation.

Three different plasmid replicon types were identified in the 17 *mcr-1*-harboring isolates (Figs. 5 and 6). The most frequent plasmid replicons were IncX4 (11/17; 64.7%), IncI2 (4/17; 23.53%), and IncHI2/IncN (2/17; 11.76%), respectively (Figs. 5 and 6). The sizes of the 11 IncX4 carrying *mcr-1* plasmids were in the range 33,309–45,011 bp, whereas the 4 IncI2 carrying *mcr-1* plasmids were in the range 60,960–67,526 bp. The 2 IncHI/IncN were 270,820 bp and 273,765 bp. As shown in Fig. 5, there was high similarity among the IncX4 plasmids, although some of had different positions of the *mcr-1* gene (nos. 56511 and 59990). In contrast, IncI2 and IncHI2/IncN, each had *mcr-1* positions. We found that IncX4-type plasmids were carried on *E. coli* STs 410 (7/11;63.6%) and ST10 (1/11; 9.1%) and on *K. pneumoniae* ST15 (1/11; 9.1%), ST336 (1/11; 9.1%), and ST340 (1/11; 9.1%). IncI2 was harbored in ST3052 (1/4;25%), ST1287 (1/4;25%) in *E. coli* and in *K. pneumoniae* ST336 (2/4; 50%), whereas IncHI/IncN were carried by *E. coli* ST6721 (1/2; 50%) and the new ST (1/2; 50%)*.*

Figure 6 demonstrated diversity in the upstream and downstream coding sequences flanking *mcr-1* in each type of plasmid. The IncHI2/IncN carrying *mcr-1* plasmids showed the repressor gene *TnpA* and the conjugative transfer gene *TraN* upstream and the tellurium resistance genes downstream of *mcr-1*. The IncI2 carrying *mcr-1* plasmids revealed the same integrase gene downstream but different genes upstream. In contrast with 11 IncX4-carrying plasmids, the flanking coding sequences or genes were similar in 9 plasmids containing the gene RepB family plasmid initiator replication protein upstream (nos. 2117, 2509, 58967, 62122, 59990, 60220) and downstream (nos. 53037, 53060, 60000) or peptidyl-arginine deaminase downstream, whereas 2 IncX4 plasmids (V417 and 56511) had different genes upstream and downstream of *mcr-1* (Fig. 6). Isolates with nos. 2117, 2509, 62122, and 60220 showed high genetic organization similarity in the plasmids (Figs. 5 and 6).

Among the *mcr-1*- harboring plasmids, other antimicrobial-resistant genes (*bla*_TEM-135_ or *bla*_TEM-1B_) than *mcr-1* were detected in IncHI2/IncN (P24-5 and P36.8) that are first reported in this study. All IncX4 and IncI2 plasmids contained only *mcr-1*; no other antimicrobial resistant genes were detected.

**Figure 7:**
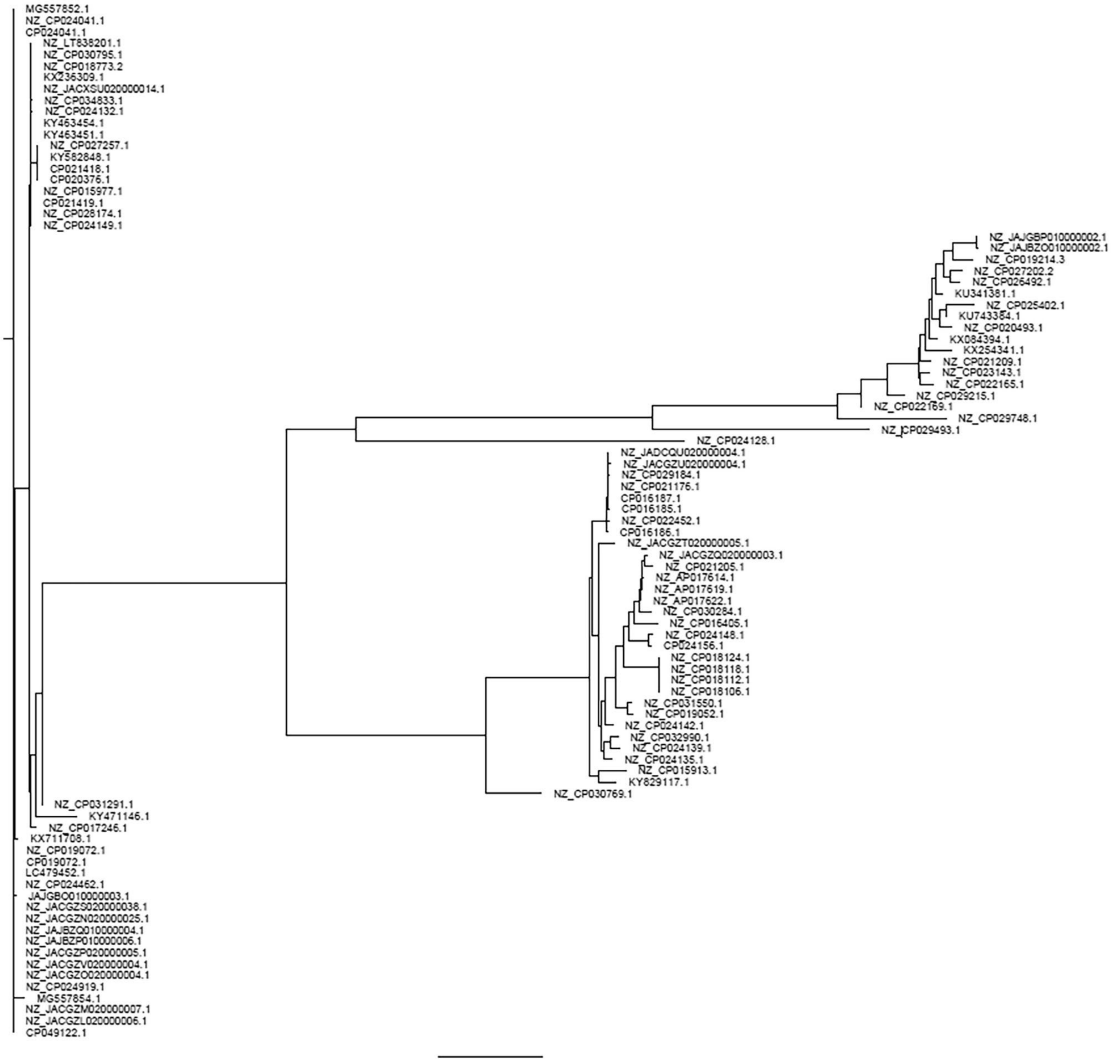
Phylogenetic analysis of *mcr-1*-carrying plasmid from whole-genome sequence in this study plus other references conducted using the Mashtree program.

As shown in Fig. 7, plasmid phylogeny demonstrated diversity among the *mcr-1* harboring-plasmids. Most IncX4 carrying *mcr-1* plasmid were grouped in three clusters. The first cluster comprised strain 59990 which was related to the pTB602 plasmid of *Salmonella* sp. SSDFZ69 from China (NZ_CP034833.1). The second cluster contained strains 58967, 50220, 2509, 62122, and 2117 that related to the plasmids pKPNH54.3 (NZ_CP024919.1) of *K. pneumoniae* NH54, and PN42 (MG557854.1) of *E. coli* PN42, both from Thailand. The third cluster consisted of 53360 and 53037 which related to the plasmid pEC931 (CP049122.1) of *E. coli* EC931 from China. We found that the other IncX4-type plasmids carrying *mcr-1* of strains 56511, 60000, and V417 were not clustered and were located on different branches (Fig. 7).

The IncHI2/IncN harboring *mcr-1* plasmids of strains P24-5 and P36.8 were clustered together with plasmids pMCR_WCHEC050613 of *E. coli* and WCHEC050613 from China (NZ_CP019214.3). Among the IncI2 type plasmids harboring the *mcr-1*, strain 54881 was related to the p1002-MCR-1 plasmid (NZ_CP021205.1) of *E. coli* Z1002 from China, whereas it was a distant relative from strains 2514-18, 61843, and 54715. However, strains 2514-18 and 1843 were grouped together with two plasmids (p5CRE51-MCR-1 of *E. coli* 5CRE51 from Taiwan (NZ_CP021176.1) and pMCR-H9 (NZ_CP029184.1) of *E. coli* H9Ecoli from China).

## Discussion

The high prevalence of human Enterobacterales isolates harboring *mcr* genes is of global concern. A recent report revealed the overall average prevalence of *mcr* genes was 4.7% (0.1–9.3%) in 47 countries across 6 continents^4^; as many as 10 variants of the *mcr* genes (*mcr-1* through to *mcr-10)* have been documented^7,8^. A recent study reported 1.03% and 0.12% *mcr*-harboring carbapenem-resistant *E. coli* and *K. pneumoniae*, respectively^13^. Up to the present, 15 Inc-type *mcr-1*-carrying plasmids have been documented, consisting of IncFII, IncHI1, IncHI2, IncI2, IncP1, IncX4, IncY, IncF, IncK, IncFIB, IncI1-1Y, IncN, IncFIIs, IncO111, and syncretic^35^. Most plasmids carrying *mcr-1* are transferable and IncX4, IncHI2, and IncI2 are predominant worldwide^34–38^. In the current study, *mcr-1* was located on 3 different plasmids (IncX4, IncI2, and IncHI/IncN), mainly on IncX4 and IncI2 that was concordant with previous reporting^39^. Our single stain of *K. pneumoniae* carrying *mcr-1* on the IncX4 plasmid was genetically almost identical to the *mcr-1*-carrying IncX4 plasmid pMCR_WCHEC1618 recovered from *K. pneumoniae* in healthy adults^40^. According to several reports in Thailand, the major plasmid types carrying *mcr-1* in Enterobacterales isolates were IncX4 and IncI2, although other plasmid replicons have been documented, including IncI, IncFIB, IncFrepB, IncY^13,40–44^. These results suggested that IncX4 and IncI2 bearing *mcr-1* mediated major transmission of colistin resistance in Enterobacterales in Thailand.

The conjugation experiment in the current study revealed that all *mcr-1*-harboring plasmids were successfully transferred from the donor to the *E. coli* recipient; according to the plasmid Inc types, they are conjugative plasmids^39^. Among such plasmids in our study, the IncX4 and IncI2 plasmid types were genetically similar, with the least variability, whereas the IncHI2/IncN plasmid type was divergent due to the fact that this type of double-Inc type plasmid contains multiple antimicrobial-resistant genes. This was consistent with the results of the two plasmids merging to perhaps increase the range of host species, plasmid fitness, and/or the acquisition of multiple antimicrobial-resistant genes^45^. Another study demonstrated that IncHI2-type plasmids are genetically divergent due to containing an MDR region which comprises a variable combination of antimicrobial-resistance genes and insertion sequences, such as Tn*6330*, in the IncHI2 type that is still highly active and is often transposable^37^. Our IncHI2/IncN plasmid also showed multiple antimicrobial-resistant genes. In addition, *mcr-1* was stably located on IncX4 and IncI2 without cut-paste transposition^37^, which could explain that why *mcr-1* was commonly distributed in these plasmids.

Plasmid phylogenetic analysis in the current study showed that most of our IncX4-type plasmids carrying *mcr-1* were grouped, although some were diverse. Notably, 5 isolates carrying *mcr-1* on the IncX4-type plasmid were clustered with *mcr-1*-IncX4 plasmids from either *E. coli* or *K. pneumoniae* from Thailand, indicating that they are close relatives and this type of plasmid is circulating in Thailand. In contrast, the other *mcr-1* plasmid replicon types in our study were mostly related to several plasmid carrying *mcr-1* types from China, perhaps suggesting that they are widely distributed in this region and they may have originated from the same source or ancestor.

The STs of *mcr-1*-harboring *E. coli* isolates in this study were mainly disseminated through local clonal expansion with a high-risk international clone ST410 that can cause several types of infection highly resistant to antibiotics and a global distribution^46^. The *mcr-1*-carrying IncX4 plasmids have also been identified in *E. coli* ST410 recovered from human blood^47^. This may suggest a possible association between *E. coli* ST410 and the carriage of *mcr-1-*IncX4 plasmids. In contrast, the most globally common ST of *E. coli* carrying *mcr-1* is ST10^4^. However, previous study in Thailand revealed the *mcr-1* carrying *E. coli* isolates from humans had diverse STs^42^. In the current study, *K. pneumoniae* ST336 was predominant. ST336 belongs to clonal complex 17, predominant in carbapenem-resistant *K. pneumoniae* and is considered an international clone frequently associated with global spread^47–50^. The *K. pneumoniae* ST15 isolates associated with the spread of multiple drug-resistance genes include ESBLs and *mcr-1*^40,48^.

*mcr-1* was widely distributed in many bacterial species such as *E. coli*, *K. pneumoniae*, *Salmonella enterica*, *Shigella* spp., *Enterobacter cloacae*, *Pseudomonas* spp., *Aeromonas* spp.*, Citrobacter freundii*, *Kluyvera ascobarta*, *Raulotella ornitholytica*, *Proteus mirabilis*, and *Acinetobacter lwoffi*^51^. *E. coli* is the most prevalent species among the *mcr*-harboring isolates, accounting for approximately 91% of the total *mcr*-carrying isolates, followed by *Salmonella enterica* (~ 7%) and *K. pneumoniae* (~ 2%)^52^. In Thailand, *mcr* harboring *E. coli* and *K. pneumoniae* has been reported approximately 1.03–2% and 0.12–1% of isolates during 2014–2019, respectively^13,53^. The *mcr* genes have been reported high prevalence rate (3.3%, 24/724) in *Salmonella* clinical isolates associated with *mcr-3* (91.67%, 22/24) and *mcr-1* (8.33, 2/24) in Thailand^54^. A previous study reported that the dissemination of 26 *mcr-1*-carrying enterobacterial strains (23 *E. coli* and 3 *K. pneumoniae*) isolated from contact surfaces (such as handrails and vending machines) on public transportation routes suggested a possible transmission vector of these organisms from one location to another, thereby posing a broader public health risk^55^. These results demonstrated that plasmids are the major vehicle involved in the dissemination of resistance or virulence genes. Notably, *mcr-1*-carrying enterobacterial strains were recovered from samples collected from hospitals in the current and the previous studies^13^, indicating that these isolates could be of nosocomial origin and thus highlighting the need for strong infection control implementation to prevent transmission of *mcr*-gene-containing bacteria capable of causing potential outbreaks.

The prevalence and dissemination of *mcr-1*-harboring Enterobacterales isolates from animals (food animals, pet animals, and wildlife), humans (healthy populations and patients) and the environment (farms, urban and rural communities, and natural environments) have been mentioned globally^36^. Control of their dissemination among humans, animals, and the environment based on the “One-health approach” is necessary. In addition, the judicious use of antibiotics is advisable to minimize the development and dissemination of colistin resistance in human isolates.

## Data Availability

The assembled genomic sequences during the current study were deposited under the BioProject with accession number JAJBZQ000000000, JAJBZP000000000, JAJGBP000000000, JAJBZO000000000, and JAJGBO000000000.
