# Publisher Correction: Plasmidome in *mcr-1* harboring carbapenem-resistant enterobacterales isolates from human in Thailand

**DOI:** 10.1038/s41598-022-26127-9

**Published:** 2022-12-13

**Authors:** Parichart Boueroy, Thidathip Wongsurawat, Piroon Jenjaroenpun, Peechanika Chopjitt, Rujirat Hatrongjit, Sathaporn Jittapalapong, Anusak Kerdsin

**Affiliations:** 1 Faculty of Public Health, Kasetsart University Chalermphrakiat Sakon Nakhon Province Campus, Sakon Nakhon, 47000 Thailand; 2 Division of Bioinformatics and Data Management for Research, Department of Research and Development, Faculty of Medicine Siriraj Hospital, Mahidol University, Bangkok, Thailand; 3 Department of General Sciences, Faculty of Science and Engineering, Kasetsart University Chalermphrakiat Sakon Nakhon Province Campus, Sakon Nakhon, 47000 Thailand; 4 Department of Parasitology, Faculty of Veterinary Medicine, Kasetsart University, Chatuchak, Bangkok, 10900 Thailand

Correction to: *Scientific Reports* 10.1038/s41598-022-21836-7, published online 09 November 2022

The Funding section in the original version of this Article was omitted. The Funding section now reads:

“This research was supported by the Kasetsart University Research and Development Institute, KURDI (R-M 52.63), Bangkok, and the Health Systems Research Institute (HSI), Thailand.”

The original Article has been corrected.

